# The ancient Z-DNA and Z-RNA specific Zα fold has evolved modern roles in immunity and transcription through the natural selection of flipons

**DOI:** 10.1098/rsos.240080

**Published:** 2024-06-19

**Authors:** Alan Herbert

**Affiliations:** ^1^ Discovery, InsideOutBio, 42 8th Street, Charlestown, MA 02129, USA

**Keywords:** immunity, transcription, evolution, Z-DNA and Z-RNA, flipons, Endogenous Retrolements

## Abstract

The Zα fold specifically binds to both Z-DNA and Z-RNA, left-handed nucleic acid structures that form under physiological conditions and are encoded by flipons. I trace the Zα fold back to unicellular organisms representing all three domains of life and to the realm of giant nucleocytoplasmic DNA viruses (NCVs). The canonical Zα fold is present in the earliest known holozoan unicellular symbiont *Capsaspora owczarzaki* and persists in vertebrates and some invertebrates, but not in plants or fungi. In metazoans, starting with porifera, Zα is incorporated into the double-stranded RNA editing enzyme ADAR and reflects an early symbiont relationship with NCV. In vertebrates, Zα is also present in ZBP1 and PKZ proteins that recognize host-derived Z-RNAs to defend against modern-day viruses. A related Zα fold, also likely to bind Z-DNA, is present in proteins thought to modulate gene expression, including a subset of prokaryote arsR proteins and the p15 (PC4) family present in algae. Other Zα variants that probably play a more general role in the reinitiation of transcription include the archaeal and human transcription factor E and the human RNA polymerase 3 subunit C proteins. The roles in immunity and transcription underlie the natural selection of flipons.

## 1. Introduction

Z-DNA is a left-handed DNA helix that is in equilibrium with the lower energy right-handed Watson and Crick B-DNA double helix. Its formation can be powered by the polymerases and helicases or other proteins that generate topological stress by the negative supercoiling, stretching or bending of B-DNA (reviewed in [1,2]). Sequences prone to flip between B- and Z-DNA conformations under physiological conditions are called flipons and typically show an alternating purine/pyrimidine motif [3]. The ease of flipping varies with sequence and decreases in the order: d(CG)_n_ > d(TG)_n_ > d(CCCG)_n_ with d(AT)_n_ still capable of forming Z-DNA, but more likely to extrude as a hairpin structure [4–7]. The alternating *syn–anti* conformation gives rise to the zig-zag backbone for which Z-DNA is named [8].

The energy cost of Z-DNA formation in a protein-free, covalently closed-circular-negatively supercoiled plasmid (cccDNA) arises from creating a junction between B-DNA and Z-DNA (BZJ) and from the adoption of the *syn* conformation by guanosines, where the base points over the sugar rather than away from it as occurs with the *anti* conformation of B-DNA bases [8,9]. In the BZJ, one base from each strand is extruded from the helix with a cost in cccDNA of 4–5 kcal mol^−1^. The cost for flipping the conformation of each d(CG) dinucleotide is 0.66 kcal mol^−1^ [10,11]. In linear perfectly paired nucleic acid, substrates that lack the torsional restraints present in cccDNA, and in the presence of different Zα binding proteins, or when there are BZJ sequence mismatches, the cost of BZJ formation may be significantly lower. The cost of BZJ formation also varies with salt concentration, as recently reviewed [12]. With some linear RNA duplexes, the increased entropy associated with bases extruded from BZJs can favour the flip to Z-RNA [13]. The increased entropy associated with RNA mismatches or bulges at the A-Z junction also facilitates Z-RNA formation [14]. However, inside cells, the long double-stranded RNA (dsRNA) substrates with the potential to elicit an interferon response are less likely to have free ends. They are bound stochastically by the helicases that trigger the downstream pathways leading to interferon production. In one model, the torsion that arises in a dsRNA fixed by helicases at either end can induce the formation of Z-RNA by Z-prone sequences [15].

Much of our understanding of the biology of Z-DNA and Z-RNA (collectively referred to as ZNA) comes from the study of the high-affinity, structure-specific Zα fold that binds ZNA and was first named in 1993, identified in 1995 and mapped in 1997 [16–18]. As predicted from the sequence, the fold is a winged helix-turn-helix domain (wHTH) [19,20]. Only two wHTH proteins in the human genome are currently known to bind Z-DNA: the interferon-induced p150 isoform of the dsRNA editing protein ADAR (adenosine deaminase RNA specific) and ZBP1 (Z-DNA binding protein 1). ADAR deaminates adenosine to form inosine [21], which is subsequently translated as guanosine [22], while ZBP1 is an interferon-induced ZNA binding protein that initiates inflammatory cell death by activating receptor interacting protein kinase 3 (RIPK3) [23]. In fishes, instead of ZBP1, there is the interferon-induced protein kinase containing Z-DNA binding domains (PKZ), where the Zα domain is associated with a PKR (protein kinase R, eukaryotic translation initiation factor 2 alpha kinase 2 encoded by EIF2AK2)-like kinase domain that regulates translational initiation and stress granule formation [24,25]. Zα paralogues are also found in a number of poxviruses, a cyprinid herpesvirus [26] and the African swine fever virus [27]. Genetic studies in humans have provided evidence that ADAR Zα loss of function variants are causal for the Aicardi Goutières Syndrome Type 6 interferonopathy, Bilateral Striatal Necrosis and Dyschromatosis Symmetrica Hereditaria [28].

**Figure 1.**
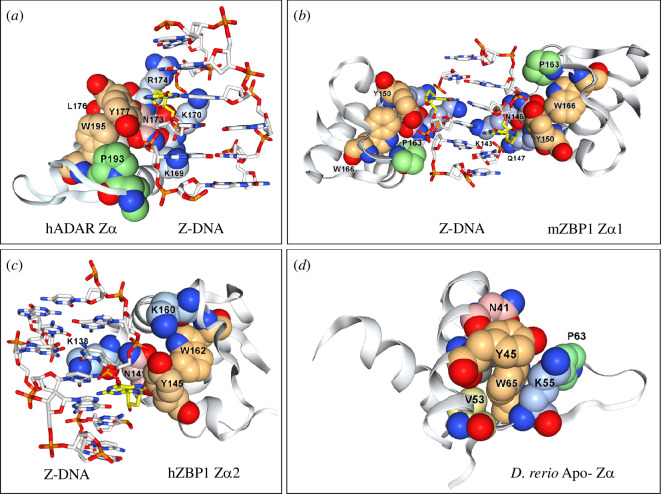
Variability of the Zα interaction with Z-DNA.(*a*-*d*) A CH-π hydrogen bond of the conserved tyrosine (Y) with C8 of guanosine in the *syn* conformation G-base (highlighted in yellow) underlies the specificity of the Zα domain for left-handed nucleic acids (ZNAs). The tyrosine is orientated by the edge-to-face interaction with the conserved Zα tryptophan. ADAR (*a*), ZBP1 Zα1 (*b*) and ZBP1 Zα2 (*c*) domains differ in the way they interact with Z-DNA and Z-RNA through proline residues, consistent with different evolutionary histories. (*d*) The structure of the *D. rerio* apo-Zα reveals how the tryptophan is prepositioned by isoleucine49, lysine51 and proline 60 to bind ZNAs (human 1QLB [19]), mZBP1.1 (mouse 1J75 [29]), hZBP1.2 (human 3EYI [30]) and *D. rerio* apo-Zα (5J6X [31]). Images created in NGL Viewer [32].

**Figure 2.**
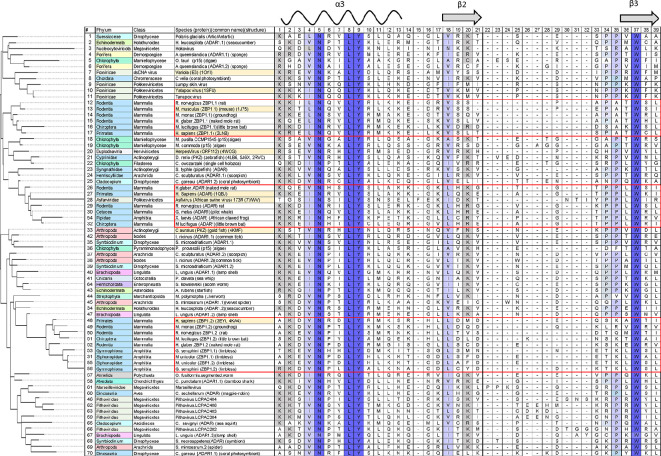
Alignment of Zα from the different domains of life. The conserved tetrad (NLYW is highlighted in dark blue). The clades are coloured by phylum (e.g. Chordata are coloured Cornish blue). The Protein Database Structure codes for solved structures are given in the ‘species’ column. The canonical wHTH Zα protein folds from clustered mammalian and amphibian ADAR and ZBP1 (with the two Zα domains labelled ZBP1. ZBP1.2) are boxed in red. In some species, there are additional copies of the ADAR gene (indicated by the name ADARx, where *x* = 1 or 2) or multiple Zα domains in a single ADAR protein (indicated by ADARx.y, where *y* = 1, 2 or 3). The location of the α3 helix and β2 and β3 strands of the wing found in ADAR Zα are indicated by the annotations at the top of the table. The β-strands within the wing are boxed. wHTH, winged helix-turn-helix. The alignments were performed using T-Coffee using the default parameters [ 33 ].

Structural studies show that Zα specifically recognizes the unusual *syn* conformation of bases in ZNA [19], reflecting the change in DNA stacking as base pairs invert during the flip from right- to the left-handed double helix (figure 1). The Zα fold is characterized by four highly conserved residues (figure 2). In human ADAR Zα, asparagine 173 (N173) makes two of its three contacts with the Z-DNA backbone through water. Tyrosine 177 (Y177) confers specificity for Z-DNA through a CH-π hydrogen bond between the aromatic ring of Y177 and the C8 of guanosine that can only occur with the base in the *syn* conformation. The tyrosine is orientated by tryptophan 195 (W195) through an edge-on-face interaction. Replacement of the tyrosine by isoleucine, as in the human ADAR Zβ domain, abrogates specific Z-DNA binding [7,34]. Replacement of the tyrosine with phenylalanine loses the indirect water-mediated hydrogen bond made by Y177 to the phosphate backbone of Z-DNA, decreasing binding affinity [19,20]. The conserved proline 193 (P193) and isoleucine 176 (I176) wedge W195 are in place (figure 1*a*). A similar fold configuration exists in the *Danio rerio* apo-Zα. Lysine 55 (K55) pre-positions tyrosine Y45 for binding to Z-DNA by hydrogen bonding to its hydroxy group, while also contributing to the W65 wedge (figure 1*d*). By contrast, the vaccinia protein E3 lacks an equivalent lysine to preposition tyrosine, only binding with high affinity to preformed Z-DNA [35]. It is also possible that the tyrosine only becomes correctly orientated in E3 dimers that form by the exchange of β-strands between monomers. Such a swap is observed in the cyprinid Zα structure [26]. ZBP1 has two Zα domains. These are numbered from the N-terminal end of the protein and called here ZBP1.1 and ZBP1.2 (corresponding to Zα1 and Zα2) to avoid confusion with other Zα domains. Both ZBP1.1 and ZBP1.2 have the conserved Zα domain residues, but interact with ZNA in a slightly different orientation: ZBP1.1 uses the P192 equivalent rather than P193, while ZBP1.2 lacks prolines within its wing (figure 2; [29,30]).

Previous studies have identified the appearance of ADAR-mediated adenosine to inosine editing early in evolution [36,37]. RNA editing events by the coral symbiont *Symbiodinium microadriaticum* and *Octopus bimaculoides* result in context-specific recoding of proteins in response to different environmental stressors [38,39]. ADAR p150 and pa110 isoforms are detectable in these and many other clades. Only the ADAR p150 variant incorporates the Zα domain, which is present at the N-terminus of the protein. It was therefore of interest to identify the evolutionary origins of the Zα fold and to examine whether it is associated with functional domains other than those currently known.

## 2. What are the evolutionary origins of Zα?

To examine these questions, a search was performed of the Prosite Sequence Database (PSD) [40] to identify proteins containing the conserved Zα ‘NLYW’ residues, starting with the ‘Nx(2)LYX[LM]X(8,20)W’ motif that was derived from the human ADAR Zα domain. The search was performed reiteratively, modifying the search motif based on the results obtained. Low stringency BLAST searches were also conducted to find additional sequences not currently in the PSD (parameters: PAM30, expected threshold = 2000, gap penalty = 8, extension penalty = 1, word size = 5, no repeat exclusion) [41]. That these sequences folded correctly into a wHTH was checked using the ALPHAFOLD2 server [42]. Additional sequences that ALPHAFOLD2 identified as having related folds were used to further inform BLAST searches. The ALPHAFOLD2 results were also used to exclude motif matches with an HTH4 conformation rather than a wHTH fold, the different outcomes were often owing to a single amino acid substitution [43]. In other examples, folds were found where the tyrosine was not aligned with tryptophan, either because the β-sheet was too short or the tryptophan was not correctly positioned in the β-sheet. The latter case is exemplified by the E2F family of transcription factors that bind to a d(CGCGCG) sequence motif that is highly prone to form Z-DNA, but in which the misaligned tryptophan lies at the carboxy-end of the β-sheet [44].

Protein sequences with folds and motifs that match Zα were then used to check for other orthologues with more distant sequence variations. Overall, Zα folds in many annotated ADAR, ZBP1, viral and PKZ genes were identified along with others that lack such annotation. A representative sample from different phyla is shown in figure 2. The results are coloured by clade and phylum. In some species, there are additional copies of the ADAR gene (indicated by the name ADARx, where *x* = 1 or 2) or multiple Zα domains in a single ADAR protein (indicated by ADARx.y where *y* = 1, 2 or 3), suggesting that ADAR is subject to different environmental selection pressures in each species. Sequences were aligned using T-Coffee as this algorithm performs well on short sequences [33,45]. The alignments are consistent with the structures shown in figure 1. The minimum length of the β-sheet is four residues, while the wing loop is quite variable. The sequence variations in the β-sheet reflect the different modes of docking of the Zα domain to ZNA.

**Figure 3.**
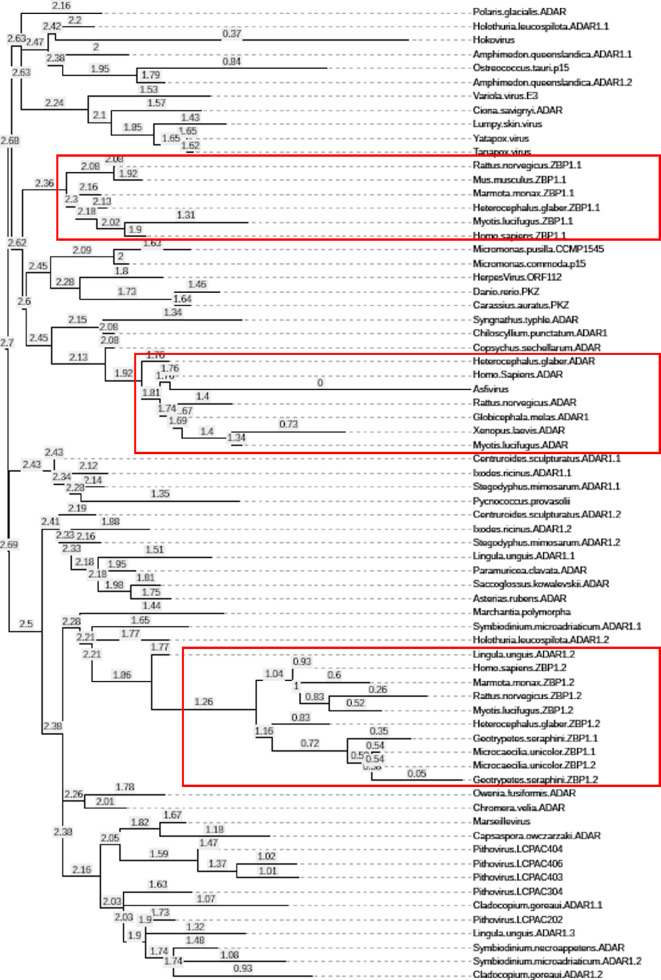
Expanded phylogenetic tree for Zα domains illustrating the evolution and relative age of the different examples detailed in figure 2 with each species named. The red boxes around mammals and amphibia highlight the different histories of the ADAR and ZBP1 domains. The tree was built by maximum likelihood using IQ-MAP [46].

The alignments were used to construct phylogenetic trees using the maximum likelihood algorithm implemented in IQ-MAP [46], with relative age for each branch given in figure 3. Overall, the different proteins are grouped together by phyla and by their presence in ADAR, ZBP1.1 or ZBP1.2. The groupings for the different Zα domains from mammals and amphibia are highlighted by the red boxes in figures 2 and 3. The phylogenetic analysis provides evidence consistent with horizontal gene transfer between clades. One interesting example is provided by the three different Zα domains present in the brachiopod *Lingula unguis* ADAR protein. Each Zα domain aligns with those from a range of unrelated species, suggesting that this brachiopod has created a Zα collage composed of the DNA it has acquired from other organisms. Similarly, horizontal gene transfer would explain the ORF112 Zα fold present in cyprinid herpesviruses that are classified as duplodnaviria based on their capsid proteins rather than as nucleocytoplasmic DNA viruses (NCV) like all the other viruses shown in figure 3 [47,48].

The Zα domains appear to have evolved earliest in the giant Hokovirus with the smaller and related poxviridae coming later. The Pithoviridae, also with smaller genomes, evolved separately and in parallel with their aquatic hosts. The most recent Zα domain is from the swine fever Asfivirus genus. Zα orthologues are also found in the earliest unicellular organisms with examples present in both archaea and eukarya. The holozoan unicellular symbiont *Capsaspora owczarzaki* is perhaps the earliest metazoan ancestor that has the Zα domain [49,50]. So far, there is no evidence for the presence of Zα in other early organisms such as Placozoa (multicellular animals without any body part or organ), Ctenophora (comb jellies) or Choanoflagellata (unicellular flagellates). Collectively the findings speak to the ancient origins of the Zα fold and support a role for *Cap. owczarzaki* early in animal evolution.

Horizontal gene transfer may also explain the evolution of the ZBP1 protein. The two Zα domains appear to have arisen separately in different branches of the phylogenetic tree (figure 3). One scenario is that the initial ZBP1 version contained only the ZBP1.2 domain that evolved from an early copy of ancestral Zα. The large sequence divergence from other Zα domains is consistent with this possibility. ZBP1.2 is closely related to and probably arose from the ADAR Zα domain of shellfish like lingula or species related to sea cucumbers. At a later time, the ZBP1.1 exon was acquired by ZBP1, probably originating from the ADAR Zα domain of animals, with which it is most closely related. Both these Zα domain variants arise in the same branch of the phylogenetic tree (figure 3). The shuttling of Zα domains between species during horizontal gene transfer probably involved NCV viruses: the giant Hokovirus is sized for uptake by phagocytosis [51], while the smaller members of the family are optimized for other modes of transmission.

## 3. Natural selection by viruses for and against Zα retention during evolution

The presence of Zα in viruses is a feature of NCV, from giant viruses to pox viruses. The giant virus arose at a time before the last common ancestor of prokaryotes, archaea and eukarya had come to dominate the future: the RNA polymerases found in all three domains are of viral origin [52]. From these early days, the evolution of NCV and their hosts appears linked as the Zα domain is found in a diverse set of clades such as prolifera, brachiopods, corals, sponges, dinoflagellates and early vertebrates as well as in metagenomic samples that uncover many poorly characterized species. The NCV acts both as symbionts and also as host virulence factors [53]. While harbouring NCV is not without cost, the advantages are many. The viruses provide a mechanism to enhance the survival of their hosts through the introduction of genetic material that encodes successful adaptations evolved by other genomes. The genes they carry can foster the production of metabolites otherwise not available to the host, while the virulence factors they produce can arm their hosts against predators and enable host parasitism of even more organisms. From a selfish gene perspective, anything viral that benefits the host benefits both.

The acquisition of Zα domains by viruses counters the host response against them. The interplay between poxvirus orthologues of ADAR and other proteins like ZBP1 is one example [54]. In turn, the Zα domains encoded by NCV may support the infectivity of amoeba and similar invasive organisms. Injection of the virally encoded proteins by a host type 4 secretion system can inhibit innate immune responses of the organism under assault [51,55]. This attack not only increases the ability of NCV-infected amoeba to cause human disease but also increases the pathogenicity of the intracellular bacteria, such as legionella, treponema and chlamydia that amoeba additionally harbour [56]. This battle involving NCV and their hosts continues today as more virulent members of the Varidnaviria and Pokkesviricetes clades continue to emerge [57].

**Figure 4.**
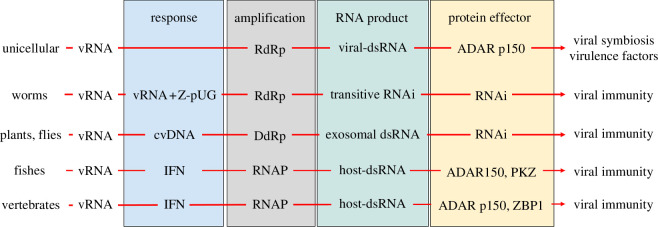
Reponses to viral RNAs differ between organisms, with viral or host-templated double-stranded RNAs (dsRNA) driving the amplification phase. vRNA, viral RNA; cvDNA, circular viral DNA;IFN, interferon; Z-pUG, Z-form poly(UG)_n_ quadruplex; RdRp, RNA-dependent RNA polymerase; DdRP, DNA-dependent RNA polymerase (type unknown); RNAi, RNA interference; RNAP, RNA Polymerase 2 (POL2).

Of equal interest is the loss of Zα orthologues in many lineages. Although found in the chloro- and streptophyta (green algae), Zα domains were discarded as other plant species became rooted in place. Canonical Zα domains have also been lost in those organisms historically used as genetic models with the intent of establishing the basic tenets of molecular biology. No examples of canonical Zα are found in nematodes, fruit flies, yeast, or Arabidopsis. Those organisms use other pathways to defend against viruses. Those responses are based on RNA-dependent RNA polymerase (RdRp) in plants and *Caenorhabditis elegans* (reviewed in [58]) and DNA-dependent RNA polymerases (DdRp) in *Drosophila melanogaster* ([59]; figure 4). Interestingly, these responses may still retain a vestige of ZNA involvement. The *C. elegans* RdRp that provides sequence-specific immunity depends on a Z-form GU quadruplex. The uniquely shaped four-stranded structure primes the synthesis of the dsRNA substrates essential for transitive RNA interference [60]. In flies, a cccDNA template derived from the pathogen is required for the DdRp to make RNAs that specifically target the threat [59]. The Z-DNA induced by the negative supercoiling that arises during template transcription [61] may provide a mechanism to localize the DdRp to these substrates. It is likely that the DdRp involved is RNA polymerase 2 (POL2), although it is currently unknown how the enzyme is targeted to the viral DNA [62]. By contrast, RdRp-dependent immunity may be disfavoured in organisms with a high retroviral load, such as primates, as the enzyme would favour the amplification and spread of endogenous retro-elements (ERE) throughout the host genome. Zα-dependent immunity may be a better option for these organisms. Of course, there are some species, such as the *Ixodes* tick, that have both Zα- and RdRp-dependent immunity. Such creatures rely on different hosts for each stage of their lifestyle: they must thwart a range of host defences regardless of whether they involve Zα or RdRp or both [63].

## 4. Natural selection of Zα domains by retroelements

In humans, the role of ADAR Zα has also evolved to counteract the invasion of our genome by retroviruses. ADAR p150 also targets other repeat elements like small interspersed nuclear elements (SINEs) that are not protein-coding but depend upon long small interspersed nuclear element (LINE) encoded factors to copy their RNA transcripts back into chromosomal DNA (reviewed in [64]). Together. SINEs, LINEs and other ERE contribute to over 45% of the human genome [65]. The SINEs preferentially insert into active genes and can disrupt their function [66]. The different waves of SINE invasion into the human genome therefore produced an existential threat to humans and probably provided strong negative selective pressure against the preservation of RdRp-mediated immune defences. The evolution by the host of proteins, like the zinc-finger proteins that define the Krüppel-associated box family, represents one way to repress ERE expression. This solution takes time to evolve [67]. The clustering of these proteins into distinct chromosomal loci provided a more rapid means to diversify their specificity by enabling trans-splicing of RNAs produced from the genes of different family members. Such splices appear to use RNA sequence motifs similar to those DNA motifs used in immunoglobin gene rearrangement [68]. Neither gene duplication nor trans-splicing guarantees protection against newly emergent retroelements.

The sensing of ERE RNAs by Zα domains provides a more general and direct strategy to protect against viral attack. There is no need for RdRp-dependent amplification of viral templates. Coupling cellular dysfunction to the expression of host ERE that form Z-RNA generalizes this solution to many diverse threats: the list includes pathogens, regardless of whether they are old or new or ill-defined, as well as to stressed or damaged or malignant cells. Interestingly, SINEs called ALUs contain a Z-BOX sequence capable of forming Z-RNA when ALU inverted repeats fold back on themselves to form dsRNA [69]. Recognition of the Z-BOX encoded Z-RNAs by Zα localizes ADAR p150 to these transcripts [14,69] and is a direct mechanism to calibrate host responses to diverse threats. Through such interactions, the host can use ADAR p150 to identify and destabilize, disrupt or inactivate EREs through RNA editing and moderate their expression and retrotransposition [28,69–73]. The strategy is quite nuanced as both EREs and the host evolve in tandem. For example, the SINE sequences that encode Z-BOXes are also prone to form Z-DNA and promote ERE expression by maintaining these elements within regions of open chromatin. The host can then adapt the leaky expression of SINE elements to its own advantage. Indeed, the presence of SINEs in host RNAs provides a mechanism to recognize such transcripts as self since the EREs are not encoded by viruses. The sensing by ADAR p150 of Z-RNA formed by SINEs thereby provides a mechanism to terminate the interferon response generated against normal cellular RNAs [15]. ADAR acts in a number of ways to regulate outcomes; the enzyme masks the dsRNA regions that otherwise activate both interferon induced with helicase C domain 1 (IFIH1)-dependent interferon responses and PKR-mediated shutdown of translation; the deaminase domain edits the dsRNA stems of transcripts; the p150 isoform Zα domain sequesters ZNAs to inhibit ZBP1 activation of inflammatory pathways [70]. Only when ADAR p150 is overwhelmed does ZBP1 trigger the immune system to eliminate threats by terminating cells. The relative activities of ADAR p150 and ZBP1 then set the threshold for either suppression or amplification of the immune response. This outcome depends only on host-encoded RNAs and not the specific recognition or RdRp-dependent amplification of pathogen sequences. The same mechanism can be also used to protect against cancers when disruption of normal gene regulation leads to rampant ERE expression. This strategy optimizes for the survival of the host and provides a rationale for the positive selection of the Z-flipons encoding ALU Z-BOX sequences as observed in higher primates [74].

## 5. Zα-linked domains

The findings provide insight into the role long played by ZNAs in ensuring the survival of both host and viruses. In this struggle of point and counterpoint, the Zα domain is associated with a number of different functional domains and motifs, such as the dsRNA binding (DRBD) and deaminase domains present in ADAR, the kinase domain present in PKZ and the RIP homotypic interaction motif that enables the activation of RIPK1 and RIPK3 by ZBP1. Viruses also combine the Zα domain with others, such as the DRBD in pox viruses. In addition to inhibiting ZBP1 via the Zα domain, vaccinia E3 modulates activation of PKR by dsRNA through its DRBD, preventing both the shutdown of translation and the formation of stress granules that otherwise restrict the virus [75,76]. Cyprinid fishes have converged to a similar strategy through the fusion of Zα to a PKR-related kinase domain to form PKZ [24]. These host responses are countered by a cyprinid herpesvirus that encodes a Zα domain within the ORF112 protein (figure 2; [25,77]).

## 6. Zα-related domains and response-specific transcription factors

The Z-DNA forming elements in repeats also help promote open chromatin and transcription of nearby sequences. Indeed, ALU expression is not only enhanced by Z-BOX sequences but also by the many transcription factor binding sites they have accumulated as they have spread throughout the genome. Those for retinoic acid and oestrogen have been well characterized experimentally [78–80]. In these cases, ALU expression can then be regulated by POL2 [81]. ALU elements can also engage and undergo transcription by POL3, mirroring their relationship to the POL3 transcribed 7SL non-coding signal peptide RNA that is involved in the transport from the ribosome of newly synthesized peptides to the endoplasmic reticulum. The level of POL3-driven ALU expression can vary according to context [65,82–84].

**Figure 5.**
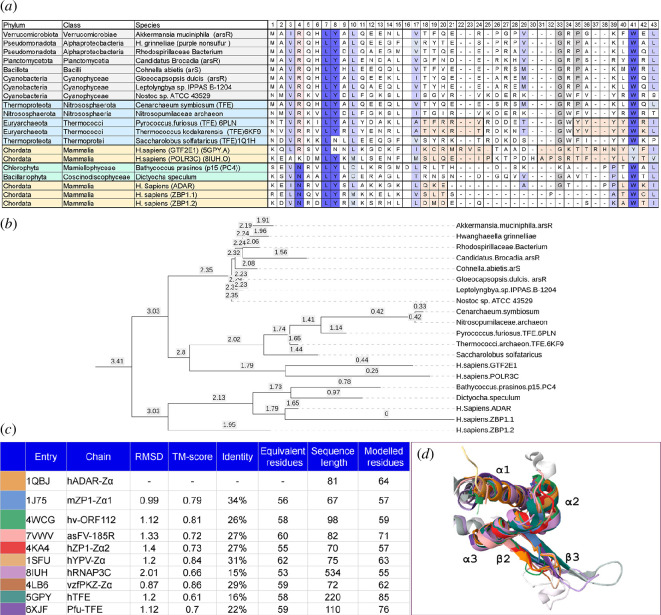
Conservation of Zα related folds across the three domains. (*a*) Alignment of human (tan), picoplankton and diatoms (green), bacterial (grey) and archaeal (blue) sequences. The alignment was performed using T-Coffee using a PAM substitution matrix [33] with adjustment of the human ADAR to align the β1 sheet residues with those of ZBP1.1 and ZBP1.2 to match that of figure 2. The bisque colouring highlights the β sheet wing residues that are defined by the crystal coordinates. (*b*) The maximum-likelihood phylogenetic tree derived using IQ-MAP [46]: showing the relative age of each branch. (*c,d*) Overlap of Zα structures with archaeal transcription Factor E (*P. furiosis*), h RNAP3C and hGTF2E1 with model parameters given in the table. h, human; zf, zebrafish; asFV, African swine fever virus; YPB, Yatapox virus; hv, herpes virus; Pfu, *P. furiosis*; TFE, transcription factor E; GTFF2E1, general transcription factor 2Eα.;TM, template modelling score, range 0–1).; PAM, point accepted mutation.

Additional roles for Z-DNA formation in transcription have received considerable support over the years [68,85–89], even though no sequence-specific, high-affinity transcription factors are currently known to bind Z-DNA. Interestingly, the Zα fold of chlorophyta (green algae) is associated with the p15 (PC4) family of domains that modulate transcription and replication ([90]; figure 5*a*). The close relationship of the chlorophyte and canonical Zα domains is evident in the phylogenetic tree presented in figure 5*b*.

Additional evidence for a link between Zα domains and transcription is provided by a subset of transcription factors from the arsR (arsenic) family (figure 5*a*). These proteins retain the canonical Zα domain leucine, tyrosine, and tryptophan residues but the asparagine undergoes a conservative arginine substitution. The effects of replacing asparagine with arginine on Zα binding to ZNA have not been explored experimentally. It is expected that arginine will bind tightly to ZNAs without the need for the water-mediated contacts that are required by asparagine. The arsR wing is extended compared to the canonical Zα domain and probably forms a longer β-sheet.

The arsR protein was first identified as a trans-regulator of bacterial responses to arsenic, providing an example of the way organisms sense and respond to environmental toxins [91]. The effects of arsenic are pleiotropic, ranging from oxidative DNA damage to altered DNA methylation to effects on iron–sulphur clusters [92]. Of course, it is possible that the arsenite can directly induce Z-DNA formation, as seen when other highly valent metals bond with the N7 of purines [93,94]. Currently, it is not known whether arsenite can induce Z-DNA. However, arsenite can induce Z-RNA [95]. In some metazoa, the oxidative stress generated by arsenite is sufficient to induce stress granules and trigger Z-RNA-dependent immune responses similar to those induced during viral infection [96,97]. Whether any of the pathways activated are involved in the efflux of heavy metals from cells is currently not reported. It is of interest to further explore the role of ZNA in responses to other environmental perturbations. The flip of B- to Z-DNA occurs quickly and provides a mechanism for a rapid response to the threats posed.

## 7. Zα-related domains and general transcription factors

Other proteins were identified that hint at less targeted roles for Zα-related domains in transcription (figure 5). These were discovered in archaea and eukarya by using search motifs that allow for more extended wings similar to those found in bacterial arsR proteins. Using this strategy, Zα-related domains were found in the archaeal transcription factor E (TFE), the human transcription factor TFEα (GTF2E1) and the human RNA POLR3C (figure 5*a*), all of which share a common ancestry [98]. These proteins are classified as general transcription factors (GTFs). They directly engage RNA polymerase and impact the transcription of many different genes.

Although the GTFs identified are related, differences have emerged as the different domains of life have diverged. The TFE from the hyperthermophiles *Pyrococcus furiosis*, *Thermococcus kodakarensis* and *Saccharolobus solfataricus*, all of which grow optimally at temperatures above 80°C [99–103], are composed of a single chain while those of eukaryotes are dimers with an α and β chain (encoded by GTF2E1 and GTF2E2) [104,105]. The phylogenetic tree reveals that archaeal and eukaryal TFE, GTF2E1 and POLR3C proteins arose from a single branch that is distinct from, but of similar age, to the canonical Zα domain (figure 5*b*).

Like the bacterial arsR, the α3 helix of archaeal and eukaryal GTFs has features similar to those of the Zα domain. The archaeal TFE maintains three of the four canonical Z-DNA binding residues (leucine, tyrosine and tryptophan), but like the arsR domain, substitutes an arginine for asparagine. The human GTF also aligns well, although GTF2E1 additionally replaces the conserved tyrosine in α3 with asparagine (figure 5*a*). Like arsR, the GTF variants all have an extended Zα wing. Indeed, the crystal structure of TFE from the hyperthermophile *P. furiosis* reveals that the β-sheet has seven bases. Interactions of the sheet with DNA are probably furthered by the four tyrosines that lie adjacent to the tryptophan and are capable of a variety of interactions with Z-DNA, as discussed above for Zα. These include the Z-DNA-specific recognition of purines in the *syn* conformation by tyrosine through CH-π hydrogen bonds to their C8 position. In both human POLR3C and GTF2E1, the conserved Zα tryptophan in the wing is replaced by tyrosine that is embedded in a patch of hydrophobic residues.

To assess the impact of the amino acid changes on the protein fold, the available crystal data were used to align the GTFs with a set of canonical Zα domains (figure 5*c*). The analysis provides an assessment of how well the folds overlap (figure 5*c*). Despite the great evolutionary separation and sequence variation, the root-mean-squared difference (rms) for 9 of the 10 structures varied only by between 0.9 and 1.4 Å. The 2.01 Å rms for RNAP3C probably reflects the variability of the loop residues within the wing. Most noticeably, the central Zα wHTH fold was preserved in all these proteins (figure 5*c*).

## 8. Modelling of general transcription factor binding to Z-DNA

**Figure 6.**
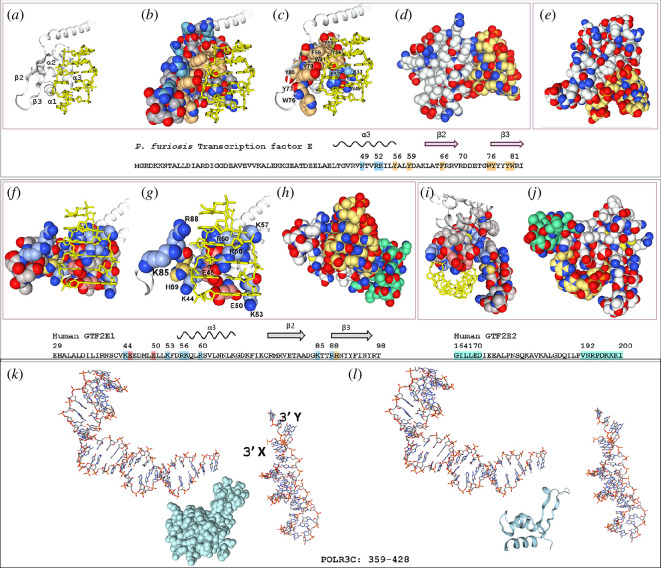
Proposed MDOCKpp [106] model of Z-DNA docked to general TFE. Alignment of *Pyrococcus furiosis* TFE wHTH and Z-DNA. The panels (*a–d*) show different views of the complex that highlight the secondary structure, the docking mode, the amino acid contacts and a space-filling rendition based on TFE amino acid residues 20–88. Panel (*e*) provides a top-down view of the tight Z-DNA docking to protein (MDOCkpp parameters; receptor: 6pln.pdb; ligand: 3p4j.pdb; clustering: yes; cut-off: 5.0; receptor blocked residues: no; ligand blocked residues: no). (*f–h*) Proposed model for docking of *H. sapiens* GTF2E1 wHTH [104] to Z-DNA. Residues 1–43 are removed during modelling as these occlude the proposed Z-DNA binding site (see figure 7). These residues are involved in docking GTFE2E1 to RNA polymerase and the switch that cycles the complex between an open and closed conformation [103]. (*f*) Side view of the interaction. (*g*) DNA contact residues and positions from (*f*)are highlighted and named. The sequences and named residues are highlighted below the images. In (*h*), amino acid residues 40–90 are presented as a space-filling model and are coloured white. Additional residues from the non-homologous GTF2E2 wHTH2 that stabilize these interactions are shaded green (amino acids 164–170 and 192–200. (*i, j*) A top-down view of the interactions. Only the GTF2E1 residues are present in (*i*), while the space-filling model in (*j*) reveals how the two GTF2E subunits of the complex create a claw to bind Z-DNA. (MDOCkpp parameters; receptor: 5gpy.pdb; ligand: 3p4j.pdb; and others as given above). (*k, l*) Position of a space-fill and cartoon model of the POLR3C wHTH relative to the 81-mer X and Y DNA chains used in the crystallization (8IUH [107]). The models from MDOCKpp are given as PDB files in electronic supplementary material.

To evaluate whether either the archaeal or human TFE could bind Z-DNA, docking studies using the MDOCKpp webserver [106] were undertaken to assess the fit (figure 6). The search was conducted using the default parameters (see figure 6 legend for details), without any further manual manipulation. The docking of archaeal pyrococcus TFE is shown in figure 6*a–c*. The different views show the specific protein contacts made with Z-DNA. The results are consistent with a cognate interaction. Space-filling models show views either from the side (figure 6*d*) or from above (figure 6*e*). The protein contact surface forms a horseshoe shape that embraces the convex surface of Z-DNA with a tight fit between the protein (in white) and Z-DNA (in yellow). The interface is enriched with tyrosines able to form CH1-π bonds with Z-DNA. The W76 and Y56 residues previously identified experimentally as DNA contacts by parabenzyoyl phenylalanine-assisted UV crosslinking [103] are close to the Z-DNA backbone and provide support for the model shown (figure 6*c*).

**Figure 7.**
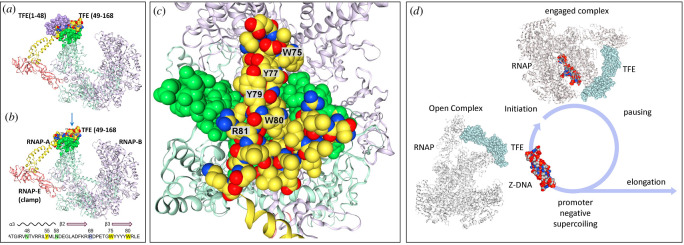
Transcription reinitiation model based on cryo-electron microscopy studies of *T. kodakarensis* [103]. (*a*) The open RNA polymerase complex shows the contact between the TFE wHTH (yellow) with the RNA-P subunit of the RNA polymerase (green). A gap that exceeds 21 Å exists between RNAP-A and RNAP-B through which DNA can pass to trigger the formation of the pre-initiation complex [103]. The TFE residues 1–48 are highlighted in blue. (*b*) The residues of TFE1-48 are removed and the arrow indicates the direction of the top-down view shown in (*c* **);** an insert shows the sequence and the position of α3 and the β-strands. (*c*) View from the top. Residues of TFE 49–168 involved in Z-DNA contacts are labelled. (*d*) Model where Z-DNA promotes transcriptional reinitiation. Negative supercoiling generated by an elongating polymerase flips Z-flipons in the promoter region to Z-DNA, which is then captured by TFE, localizing the DNA strand to the open RNA polymerase complex. As Z-DNA flips back to a right-handed conformation, the DNA double helix opens up for the RNA polymerase complex to engage the transcription start site. After an initial transcriptional burst, the polymerase enters a paused state and remains stalled until transcriptional elongation is triggered, inducing Z-DNA to reinitiate the cycle by re-engaing TFE.

Cryo-electron microscopy studies of the related *T. kodakarensis* provide information on the structural shift occurring when the open RNA polymerase complex adopts closed conformations after docking to the DNA transcription start site (figure 7). In the open complex, the gap between W75 of TFE and RNAP-B is at least 21 Å and sufficient for the passage of DNA [103]. TFE plays a pivotal role in regulating this transition to the closed form, with the shift of the TFE wHTH relative to the C-terminus mediated by the linker sequence between them. The potential Z-DNA binding residues of TFE are orientated outwards from the complex with the other face of the wHTH sitting on top of the RNAP-A subunit [103] (figure 7*c*). TFE residues 1–48 (highlighted in blue in figure 7*a*) partially cover the potential Z-DNA docking site. One possibility is these TFE amino-terminus residues modulate the interaction of the wHTH with Z-DNA, much as the aspartate and glutamate-rich region of methyl-CpG-binding domain protein 3 (MBD3) inhibits the binding of Zα to Z-DNA [108]. The structural shifts that TFE undergoes as the polymerase changes conformation may expose the residues required to engage Z-DNA.

The docking of Z-DNA to the human GTF2E1 (figure 6*f–h*) was performed using the dimer of TFEα and TFEβ and in the absence of residues TFEα residues 1–43, as these residues are equivalent to the *T. kodakarensis* residues described above that occlude the binding site (figure 7*a,b*). In the model shown, GTF2E1 is orientated towards the minor groove of Z-DNA (figure 6*i,j*). Contacts with Z-DNA also involve TFEβ, which has no sequence homology with Zα and is primarily thought to stabilize the interaction of the TFE dimer with the RNA polymerase complex [104]. The contacts that TFEβ makes with Z-DNA are highlighted in green in figure 6*j* and involve an arginine/lysine-rich patch (see sequence insert, figure 6*j*). Together, the two TFE subunits create a claw around Z-DNA, with specificity of interaction potentially arising from the insertion of residues into the Z-DNA minor groove. The arginine that replaces the asparagine in α3 directly contacts the Z-DNA backbone. Surprisingly, none of the interactions involve the aromatic amino acids of GTF2FE1, with the possible exception of H89 (figure 6*g*). Given the difference in the binding of canonical Zα and TFE, it is possible that the model of docking is incorrect. However, the variations in Z-DNA contacts between the species could reflect adaptations made to very different environments. An alternative possibility is that the archaeal TFE and GTF2E1 form complexes with Z-DNA that capture different phases of the interaction. In this scenario, the initial docking of TFE to Z-DNA may be to the convex surface of Z-DNA. The helix then rolls over the surface of the domain to unwrap the helix, opening up the transcription bubble for RNA polymerase to bind. During this process of ‘roll and unwrap’, the closed complex would then form around the bound DNA (figure 7*d*). Alternatively, the insertion of GTF2E1 residues into the minor groove of Z-DNA may ensure optimal placement of the complex by allowing the use of the backbone as a zig-zag rachet. Currently, there is no available high-resolution structural mapping of the contacts made by human GTF2E1 to DNA to validate the docking model proposed here. However, GTF2E1 is known to contact DNA, even though this has not been observed in the available crystal structures [109].

The potential interaction of POLR3C with Z-DNA is of interest as the earliest RNA polymerases are thought to trace back to NCV and most closely resembles the modern-day POL3 [52]. The crystal structure of the POL3 open complex reveals that the POLR3C Zα fold approximates the DNA helix but is not engaged. The wHTH projects into a region that lies between the overhangs of the two 81 mer base pair DNAs used in the study (figure 6*h,i*). If joined, the region between the DNAs would be severely bent. The space in between is large enough to accommodate a Z-DNA helix flanked by two B-Z junctions. Potentially the POLR3C subunit modulates the transcription of SINEs by binding to the sequences that encode the Z-BOX. Such an interaction without the engagement of POL3 may have helped to limit the many ALU invasions of the human genome during previous epochs. Now, the Z-BOX is repurposed to modulate host immune responses against such threats, both old and new. The sequence-specific recognition of viral nucleotides is now not necessary.

Interestingly, the mapping of POL3 sites in the genome reveals that approximately 30% lie within CpG islands, which are also generally enriched for Z-DNA forming sequences [68]. POL3 sites also map to POL2 promoters and enhancers [110]. The formation of Z-DNA and engagement of POLR3C to initiate POL3 transcription may therefore play a role in regulating transcription by POL2. For example, the engagement of POL3 at promoters and the formation of open chromatin may represent a way to kick-start POL2 promoters during the early stages of embryogenesis [84]. In the period immediately after the parental epigenetic marks are erased, a wave of transcription initiated at EREs upstream of the canonical gene promoters appears critical for the maintenance of pluripotency [111]. Interestingly, the persistence of pluripotency also requires POLR3G, a subunit in the POL3 complex that is bound to POLR3C. Knockdown of POLR3G reduces the transcription of around 4–5% of protein-coding genes expressed at this stage. About 30% of these genes also engage pluripotency transcription factors during this time to promote transcription by POL2 [112,113]. It is possible that POL3-induced Z-DNA formation at upstream ERE leads to the activation of the downstream POL2 promoters required for tissue-specific developmental programmes [114]. The potential role of flipons and small RNAs in these processes is described further in two recent papers [1,115].

Studies of transcription in other organisms, particularly yeast, have also been undertaken to understand the role of GTFs like TFE in the assembly of the pre-initiation complex (PIC). Results from an early biochemical analysis and from a recent study tracking transcription factor turnover confirm that TFE plays a key role in driving high rates of RNA production by promoting transcription reinitiation [116,117]. However, in yeast, the genome is AT-rich and generally does not have strong Z-DNA forming sequences upstream of the transcription start site. In this case, the interaction of TFE with DNA may involve other flipon motifs besides Z-DNA, the nature of which will require further experimentation to determine. Also, in *D. melanogaster*, the four canonical ‘NLYW’ Zα residues have been replaced by ‘RITY’ in TFIIα, probably reducing the affinity of this GTF for Z-DNA. Other mechanisms may be involved in transcriptional reinitiation in this species.

Collectively, the findings on Zα- related proteins discussed are consistent with the model presented in figure 7*d*. Here, Z-DNA formation by flipons is powered by the negative supercoiling generated by an elongating polymerase. The Z-DNA then promotes reassembly of the PIC by engaging a Z-DNA-specific wHTH domain in a factor such as TFE, priming the cycle to repeat [115]. The level of gene expression then varies throughout the genome and depends upon when and where Z-DNA formation occurs. The flip requires power. The energy needed varies with the propensity of a sequence to form Z-DNA and reflects the history of mutation, repair, recombination and retro-element mediated insertion of flipons at each locus. Sequences with lower energy requirements will promote transcription by opening up chromatin to increase the accessibility of nearby promoters. Whereas codons store information as static code, flipons modify the readout of those seqeunces by changing conformation. Consequently, changes in phenotypes require either changes in codon sequence or flipon dynamics. The former is more precise and harder to perfect, while the latter is sloppier and often just good enough for an organism to survive the moment [3].

The study has two main conclusions. First, the Zα fold is really ancient, reflecting roles played by ADAR, ZBP1 and PKZ in protecting the host. This conclusion is well-validated experimentally. Second, Zα-related proteins localize the transcription machinery to sites of Z-DNA formation. This conclusion requires additional experimental support. The involvement of ZNAs in immunity and transcription provides a rationale for the natural selection of flipons. ZNAs promote reproductive success by protecting a host against threats and by promoting the phenotypic pliability of progeny.

## Data Availability

I have listed the webservers used at the end of this section and I have given a file with accession numbers for the sequences used in the analysis. All this information is available to anyone with a web browser and there is nothing proprietary. Electronic supplementary material data accession codes for sequences in figures 2 and 5, model for P. furiosis TFE bound to Z-DNA, model for *H. sapiens* GTF2E1 bound to Z-DNA. Webservers: Blast [41]; Prosite [40]; Mdockpp [106]; Protein Data Base [118]; Structure Alignment [119]; Sequence Alignment (T-Coffee) [42]; Phylogeny Tree (IQ-Map) [46]; Phylogeny Display (ITOL) [120]; Genbank [121]; Uniprot [122]. Supplementary material is available online [123].
